# Enhancing the precision of circular stapled colorectal anastomosis: could powered stapler technology provide the solution?

**DOI:** 10.1007/s10151-019-02031-9

**Published:** 2019-07-05

**Authors:** R. Mirnezami, A. Soares, M. Chand

**Affiliations:** Department of Colorectal Surgery, University College London Hospital, 235 Euston Road, London, NW1 2BU UK

## Introduction

The use of circular stapling devices to facilitate low colorectal anastomosis (CRA) was first described in the 1970s. This approach is now standard practice and has consistently demonstrated equivalence in terms of safety and efficacy compared with hand-sewn anastomosis, with the added advantages of shorter anastomotic time and greater reproducibility. Briefly, the key elements of circular stapled CRA comprise: (1) selecting a stapler gun of appropriate size; (2) securing the anvil in the distal end of the mobilised colonic conduit; (3) performing a rendezvous manoeuvre to securely dock the anvil onto the end of the stapler gun, which has been advanced from below; and (4) firing the stapler to complete the anastomosis.

While there have been subtle modifications to staple design and configuration, there has been relatively little progress made in circular stapler functionality over the past few decades. Further advances in stapling technology could enhance the precision of CRA with the potential to positively impact on the incidence of anastomotic leakage and on the need for diverting stoma. However, refining the existing design requires the four key steps outlined above to be carefully considered to determine how and/or where the technology can be fine-tuned; sizing and anvil application are obligatory steps, while the rendezvous technique represents an elegant design that lends itself readily to both open and intra-corporeal CRA. The final and arguably most critical step in the technique, namely, discharging the stapler, represents a component in the pathway, where refinement may be both feasible and advantageous, and in this regard, the powered surgical stapler is emerging as a potential solution.

Powered surgical stapler devices were first introduced in 2011 and have been applied to a wide variety of clinical applications. More recently, powered circular stapling devices have been developed specifically intended for use in colorectal surgery. We provide a technical description of our initial experience with the ECHELON CIRCULAR™ Powered Stapler (Ethicon, Somerville, NJ, USA), which was employed during laparoscopic anterior resection carried out at a tertiary colorectal referral centre (University College London Hospital (UCLH), London, UK).

## Technical description

Laparoscopic partial mesorectal excision (PME) was performed in a previously medically fit 68-year-old, male with a biopsy proven adenocarcinoma of the upper rectum, staged with magnetic resonance imaging (MRI) as T4a N2, with no evidence of distant metastases. Dissection was performed in the standard fashion with medial-to-lateral dissection, identification, and preservation of the left ureter and gonadal vessels, proximal ligation, and division of the inferior mesenteric vein and artery, followed by PME down to the intended level of rectal transection. An extended cuff of anterior abdominal wall parietal peritoneum was excised en bloc with the specimen, where the serosal surface was breached by tumour. The splenic flexure was mobilised fully before the rectum was divided intra-corporeally using an Echelon Flex 45 stapler. A 4 cm trans-umbilical incision was used to extract the specimen and apply the anvil to the distal end of the colonic conduit in the standard fashion. A CDH 31 ECHELON CIRCULAR™ Powered Stapler was used to construct a tension-free low CRA. Anastomotic donuts were checked and confirmed to be both intact and of adequate thickness. An air-leak test was performed on table which demonstrated no evidence of leak at the anastomotic site and the anastomosis was directly visualised by flexible sigmoidoscopy, confirming its integrity. In view of the favourable circumstances and adequacy of the anastomosis, the decision was taken not to construct a diverting ileostomy. The patient made an uneventful recovery in hospital and was discharged home with a final histological stage of pT3N1.

## Discussion

A key challenge with the operation and handling of conventional, manual stapling devices is the force required to effect penetration of staples through tissue, which can be variable depending on the operative circumstances. In generating this force, the hands of the operating surgeon are prone to unwanted movements, which are further magnified along the length of the stapler shaft to the site of intended anastomosis when the stapler is being fired. These factors are believed to be, at least in part, responsible for technical errors that have been described during stapled CRA. For example, a study by Offodile et al. [1] provided data on 349 consecutive patients undergoing left-sided colon and rectal resections at a single centre and identified a circular stapled anastomotic malfunction rate of 9% (32/349 patients). Although data were comparable between the control group and patients who experienced anastomotic error, the latter had a significantly higher incidence of positive air-leak tests and proximal diversion (34% versus 16%; *p* = 0.0003).

It is logical from a tissue mechanics perspective that undue/erratic movement at the anastomotic site could result in microvascular trauma and fractures at the bowel interface, potentially compromising the anastomosis. Moreover, several previous studies have demonstrated that some surgeons are simply incapable of generating the grip-strength force required to fire circular staplers [2, 3]. Design improvements in circular stapling technology that enhance the device-to-user interface (i.e., improve handling, reduce force-to-fire requirements) whilst simultaneously addressing issues with device-to-tissue interaction (i.e., reducing unwanted movement at the anastomosis during construction) have the potential to decrease the rate of technical errors and lead to improvements in clinical outcome.

Over the past, few years powered surgical staplers have emerged on the market and these may offer a means of overcoming the force-to-fire barrier in stapled CRA. It is believed that reducing reliance on the surgeon to generate this force will help to improve the stability of the stapler during firing, potentially greatly reducing unwanted movements at the anastomotic site. It is also conceivable that the powered operation could result in more consistent compression of stapler onto tissue, potentially minimising the average compressive force experienced by target tissue compared with using a manual stapler.

Another new feature of this stapler is the staple configuration which is described as 3D. There is relatively sparse literature investigating the effect of staple configuration on anastomosis and those reports that are published are of linear stapled anastomoses. Foo et al. [4] have studied the outcomes of 340 patients and whether triple staple lines and enhanced configuration (3D) have an effect on clinical outcomes. They could not identify whether any benefit in patient morbidity was specifically associated with staple configuration, but there was an overall improvement when a stapler with triple staples and 3D configuration was used over conventional staplers. There was no significant change in the anastomotic leak rate. According to the manufacturer of the circular stapler in this report, the 3D stapling configuration and offsetting of the staple legs provide a more even compression of tissue and more optimal conditions for anastomotic healing. These benefits are in addition to the advantages of atraumatic Gripping Surface Technology that has separately been shown to provide gentler handling with a reduction in compressive forces on tissue [5].

The novel technology introduced in this stapler can confuse somewhat with automation being combined with claimed improved stapling technology. On one side, there are the obvious benefits which come with reduced movement at the time of firing. However, whether there is further optimisation of the anastomosis by the new stapling technology is more difficult to quantify or appreciate at first glance. Further clinical studies on this claim are welcomed.

Although the gains are likely to be incremental in the short term, we believe that in the longer term, we may see more significant clinical outcome improvement and hope that this will be reported in clinical studies. The powered device will compensate for differences in physical grip strength, unintentional movement, and level of experience, to create a more technically robust and reproducible anastomosis than is currently possible with manual alternatives.

## Electronic supplementary material

Below is the link to the electronic supplementary material.

Supplementary material 1 (MP4 110465 kb)
